# Platinum Carbonyl Chini Clusters as Catalysts for Photocatalytic H_2_ Generation

**DOI:** 10.1021/acs.jpcc.5c00212

**Published:** 2025-04-23

**Authors:** Aleksander Senderowski, Ana Andrea Méndez-Medrano, Isabelle Lampre, Hynd Remita, Dorota Rutkowska-Zbik

**Affiliations:** † Interdisciplinary Centre for Mathematical and Computational Modelling, University of Warsaw, ul. Adolfa Pawińskiego 5A, Warsaw 02-106, Poland; ‡ Institut de Chimie Physique, UMR 8000 CNRS, Université Paris-Saclay, Orsay 91405, France; § Jerzy Haber Institute of Catalysis and Surface Chemistry PAS, ul. Niezapominajek 8, Kraków 30-239, Poland

## Abstract

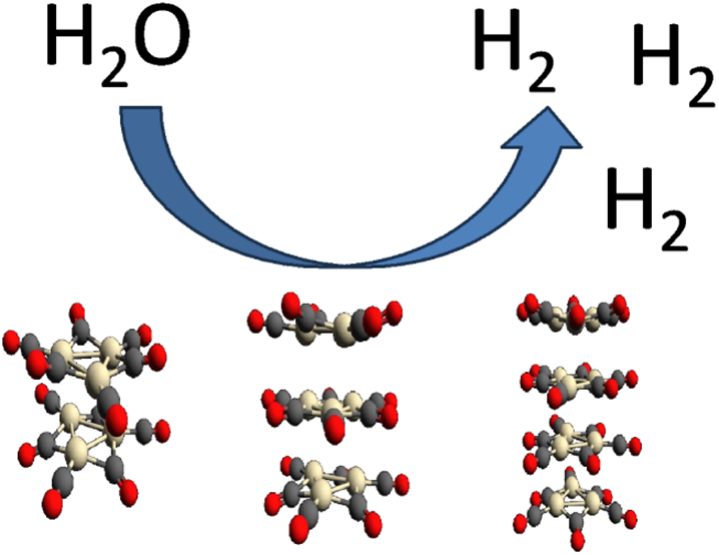

Platinum Chini clusters with a general formula [Pt_3_(CO)_6_]_*n*_^2–^ are formed by stacked Pt_3_ units. They have fascinating electronic and optical properties that can be tuned with *n*. In the current manuscript, the electronic, photochemical, and charge transport properties of the platinum Chini clusters are studied with the density functional theory (PBE and CAM-B3LYP/6-31G(d,p)+LANL2DZ) as a function of their nuclearity number *n*. Our theoretical predictions are supported by experimental proof of concept, in which synthesized Chini clusters are deposited as cocatalysts on TiO_2_ for photocatalytic hydrogen generation. We demonstrate that smaller clusters (*n* = 4) are more effective than larger ones (*n* = 7–8), and that composites having lower Pt content perform better.

## 1 Introduction

**Figure 1:**
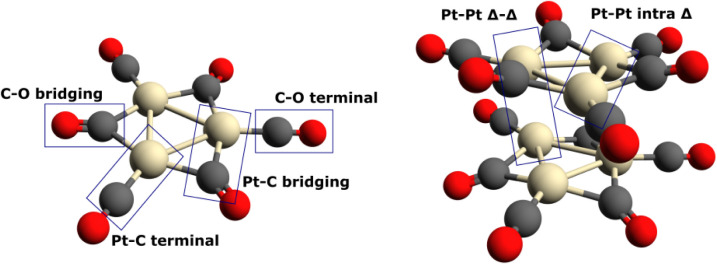
Geometries of the Chini clusters for *n* = 1 and *n* = 2 with specific bonds marked. The illustration highlights the intratriangle and intertriangle Pt–Pt distances and two types of CO ligand binding (terminal and bridging).

The synthesis of platinum carbonyl clusters with a [Pt_3_(CO)_6_]_*n*_^2–^ formula, known as Chini clusters, was proposed in the 1960s.^1^ They can be synthesized by chemical reduction by CO in an alkaline alcoholic solution. They can also be synthesized by radiolysis in alcohol solution under a CO atmosphere, where the nucleation number *n* can be tuned by the irradiation dose.^2,3^ They consist of multiple stacking units of Pt_3_ triangles stabilized by CO ligands bound in two different ways: terminal (connected to one Pt atom) and bridging (connected to two Pt atoms) – see Figure 1. Their physicochemical and optical properties depend on the nuclearity number *n*. Their redox properties have been studied by pulse radiolysis.^3^ They serve as precursors for Pt nanoparticles and nanowires, substrates to obtain metal–organic frameworks,^4^ or even alloy nanoclusters when mixed with other metals.^2,5−9^ The Chini clusters can be deposited on different supports to be used as composite materials in several electrocatalytic applications^10,11^ and photo- and thermocatalytic processes.^12−16^

Platinum is known as a very active cocatalyst for hydrogen generation, ascribable to its higher work function, extremely low required overpotential, and optimal hydrogen adsorption-free energy.^17−19^ Most of the studies on this topic report on the use of Pt-based nanoparticles for photocatalytic hydrogen formation; however, articles on clusters of controlled size as cocatalysts are scarce. Here, we aim to check the applicability of carbonyl Pt clusters in photocatalytic hydrogen generation by determining their electronic, photochemical, and charge transport properties as a function of their nuclearity. By assuming that the reaction involves the formation of H_2_ via the recombination of two hydrogen atoms, we focus on the binding of an atomic hydrogen and an H_2_ molecule by the Chini clusters of *n* = 1–8. Our theoretical predictions are followed by an experimental proof of concept, in which synthesized Chini clusters are deposited on TiO_2_ and such a composite is tested in photocatalytic hydrogen generation.

## 2 Methodology

### 2.1 Theoretical Methods

To determine the geometry and electronic properties of the Chini clusters, quantum chemical calculations within density functional theory (DFT) and time-dependent density functional theory (TD-DFT) were performed.

Geometries were optimized using the PBE functional,^20−22^ which proved to be the right choice for this class of compounds,^23^ while the investigation of excited states was conducted with the CAM-B3LYP hybrid functional.^24^ The latter better describes long-range charge transfer states and overcomes issues of convergence and computation time. The 6-31G(d,p) basis sets for C, O, and H,^25,26^ as well as LANL2DZ pseudopotentials for Pt, were implemented.^27−29^ The calculations were performed in vacuum using the Gaussian 16 software.^30^

To characterize the charge transfer properties of the studied systems, we invoked the Marcus model.^31−35^ According to the theory, the rate of charge transfer is given by:
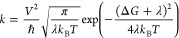
1where *V* is the electronic coupling matrix element (mostly the orbital overlap), which should be maximized for the most effective transport, *T* is the temperature, *k*_B_ is the Boltzmann constant, Δ*G* is the driving force for the charge transfer, and λ represents the reorganization energy due to the structural relaxation accompanying charge transfer. The latter parameter is composed of two elements: the external reorganization energy, representing the effect in the surrounding medium (usually the solvent), and the internal reorganization energy, related to the magnitude of the energy change due to structural relaxation while changing from the initial to the ±1 charged molecular state and vice versa. λ should be as small as possible to ensure fast transfer of charges, both holes and electrons. In the current work, we focus on the internal relaxation energy, as the external part is often difficult to evaluate with theoretical methods.^36^ Therefore, hole (λ_h_) and electron (λ_e_) reorganization energies were calculated using the following formulas:
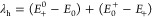
2
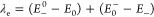
3where *E*_0_, *E*_–_, and *E*_+_ refer to the energies of relaxed molecules: neutral (*E*_0_), with an additional electron (*E*_–_), or with an additional hole (*E*_+_);  and  refer to the energies of molecules with an additional electron or hole in the geometry of a neutral state, respectively;  and  refer to the energies of neutral molecules in the geometry of a state with an additional electron or hole. A visual representation of these energy levels is shown in Figure S1.

The binding energies (BE), enthalpies (Δ*H*), and Gibbs free energies (Δ*G*) for the adsorption of the hydrogen species (H and H_2_) on [Pt_3_(CO)_6_]_*n*_^2–^ were calculated using the following reactions (for the H atom and the H_2_ molecule, respectively):
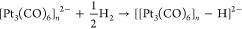

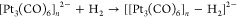
as a difference between the enthalpies/Gibbs free energies of the products and substrates. The calculations were performed using the PBE functional with Grimme’s dispersion correction with Becke-Johnson damping,^37^ and the 6-31G(d,p) basis sets for C, O, and H and the LANL2DZ pseudopotentials for Pt, as described above. The enthalpy/Gibbs free energy corrections to the electronic energies were computed at 298.15 K, as described in ref (38).

### 2.2 Synthesis of Pt Carbonyl Clusters and Their Deposition on TiO_2_

All the chemicals were used as received: platinum(II) bis(acetylacetonate) (Pt(acac)_2_, Aldrich, 99% purity), TiO_2_ (P25, Evonik), ethanol, HCl, and NaOH. Argon (Ar) and carbon monoxide (CO) gases were obtained from Air Liquide. Deionized water was generated using a Milli-Q system (18.2 MΩ·cm).

A platinum precursor Pt(acac)_2_ was dissolved in ethanol to obtain a solution with a concentration of 10^–3^ M. The pH was measured and controlled by adding droplets of aqueous solutions of NaOH and HCl, since the pH control is essential to achieve the desired target molecules.^3^ It is known that a strongly basic environment (pH = ∼13) results in smaller clusters (*n* = 4), whereas a neutral pH (pH = 7) favors the formation of larger clusters (*n* = 7 or 8). The obtained solutions were deaerated with argon gas for 20 min and subsequently saturated with CO (1 atm). Next, the solutions were irradiated with a ^60^Co panoramic gamma source for 20–40 min (the total dose of 835 Gy, low-dose irradiation). UV–vis absorption spectra were recorded by using a single-beam Hewlett-Packard 8453 spectrophotometer and 1 cm optical path cuvettes.

The commercial TiO_2_–P25 was used as a support to obtain Chini–TiO_2_ composites having 0.1 and 0.02 wt % of clusters via an impregnation method. Adjusted volumes of a previously irradiated cluster solution (1.0 and 0.2 mL, 10^–3^ M Pt concentration) were added to the weighed TiO_2_–P25 (∼195 mg) and diluted with ethanol to give 8 mL suspensions. The ethanol suspensions of the Chini clusters and TiO_2_ were covered with aluminum foil and stirred continuously overnight. Then, the obtained photocatalysts were centrifuged, washed with ethanol, and dried in an oven (60 °C, 24 h) to obtain dry powders.

### 2.3 Photocatalytic Activity for Hydrogen Generation

The resulting powders were tested for photocatalytic H_2_ generation. The photocatalyst (20 mg) was placed into a photoreactor. 15 mL of water and 5 mL of methanol (which plays a role of a hole scavenger) were added to form a heterogeneous mixture. The prepared suspensions were degassed in the dark for 20 min under an Ar atmosphere with continuous stirring. Subsequently, they were irradiated using a 300 W xenon lamp, generating a collimated beam with a continuous spectrum ranging from UV to near-infrared (250–2000 nm). The amount of H_2_ produced over time from the water–methanol solution was measured using gas chromatography (GC) with an INFICON Micro GC Fusion Gas Analyzer.

## 3 Results and Discussion

### 3.1 Geometry

The geometries of the Chini clusters for *n* = 1–8 were determined – see Table 1 and Figure 1 for the explanation of the notation used therein, explained with the examples of [Pt_3_(CO)_6_]^2–^ and [Pt_3_(CO)_6_]_2_^2–^. Figure S2 illustrates the remaining Chini clusters for *n* = 3–8. While different conformers (e.g., “wires”, “zigzags”, and others) are possible,^7^ “wires” are found to be energetically favorable, in agreement with previous studies.^5,9^ As stated in the Introduction section, in the Chini clusters, two different types of the CO ligands are present: terminal and bridging. The length of the C–O bonds varies between 1.163 and 1.204 Å, depending on the cluster size and the type of carbonyl ligand. For the terminal bonds, both C–O and Pt–C bonds are slightly shorter (by around 0.02 Å) compared with the bridging ones. The Pt–C bond length ranges from 1.864 to 2.109 Å. The Pt–Pt bond lengths and angles slightly differ, primarily between internal and external Pt_3_ units. Subsequent Pt_3_ units are rotated by approximately 16–33°. The intratriangle Pt–Pt distance ranges from 2.752 to 2.770 Å, while the intertriangle (Δ−Δ) Pt–Pt distance varies between 3.136 and 3.200 Å. The obtained geometry parameters are in good agreement with the X-ray structures and previous theoretical calculations.^39^

**Table 1:** Interatomic Distances (in Å) and Dihedral Angles (°) for the Chini Clusters (*n* = 1–8) Computed at PBE+D3/6-31G(d,p) for C, O, and H, and LANL2DZ for Pt

	Bond length [Å]						Dihedral angle [°]
	Pt–Pt		Pt–C		C–O		
*n*	intra Δ	Δ−Δ	terminal	bridging	terminal	bridging	Pt–Pt–Pt–Pt
1	2.752	-	1.864	2.079	1.187	1.204	-
2	2.761–2.763	3.137	1.874	2.080–2.097	1.175	1.193	32.8
3	2.748–2.767	3.136–3.140	1.878–1.885	2.085–2.103	1.169–1.171	1.186–1.189	20.2–20.2
4	2.752–2.770	3.142–3.155	1.881–1.889	2.088–2.105	1.167–1.169	1.184–1.187	16.7–22.8
5	2.754–2.770	3.153–3.172	1.883–1.893	2.090–2.108	1.165–1.167	1.183–1.186	16.7–21.2
6	2.755–2.769	3.163–3.181	1.884–1.894	2.091–2.108	1.164–1.167	1.182–1.185	16.5–21.4
7	2.755–2.769	3.172–3.192	1.885–1.896	2.092–2.108	1.164–1.166	1.181–1.185	16.8–21.7
8	2.756–2.768	3.175–3.200	1.886–1.897	2.093–2.109	1.163–1.165	1.181–1.184	17.2–22.0
*n*↑	?	↑	↑	↑	↓	↓	?

We noticed that with a growing cluster size, the Pt–C bond lengths tend to increase, while the C–O bond lengths tend to decrease for both types of ligands (terminal and bridging). The intratriangle Pt–Pt distance increases, whereas the intertriangle Pt–Pt distance does not exhibit a consistent trend.

Further studies, including TD-DFT calculations, were performed on the described set of geometries.

### 3.2 Frontier Orbitals

**Figure 2:**
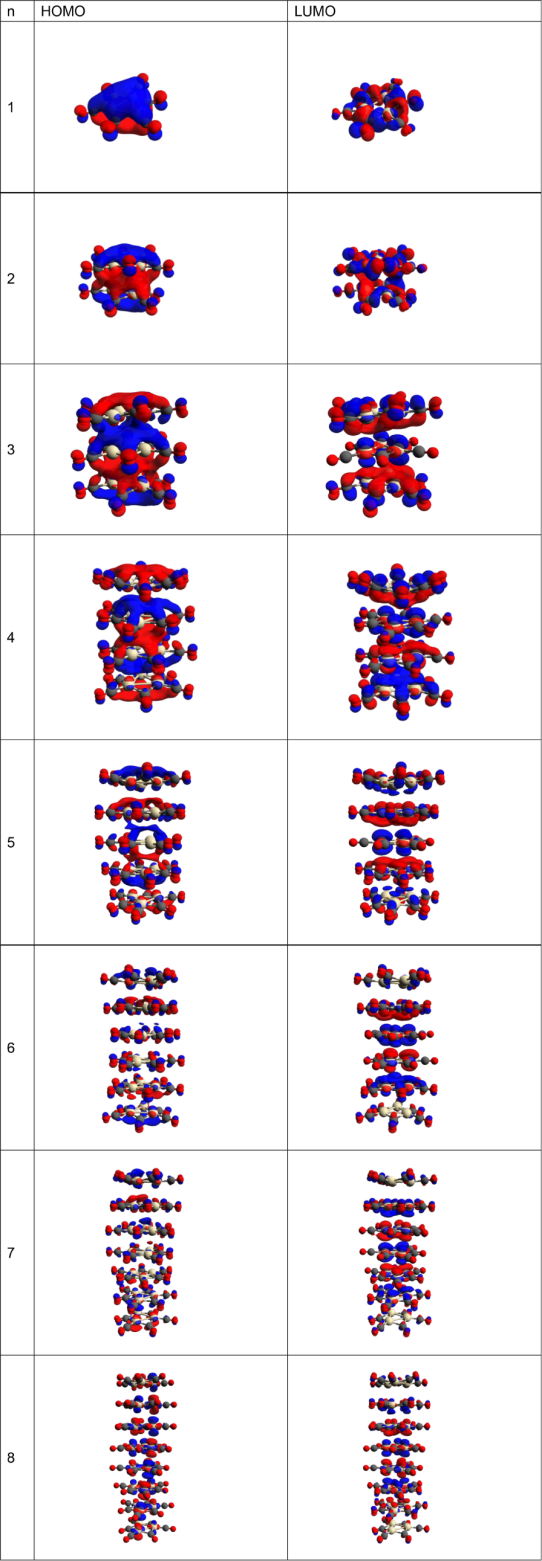
Plots of the HOMOs and LUMOs for Chini clusters (*n* = 1–8).

**Figure 3:**
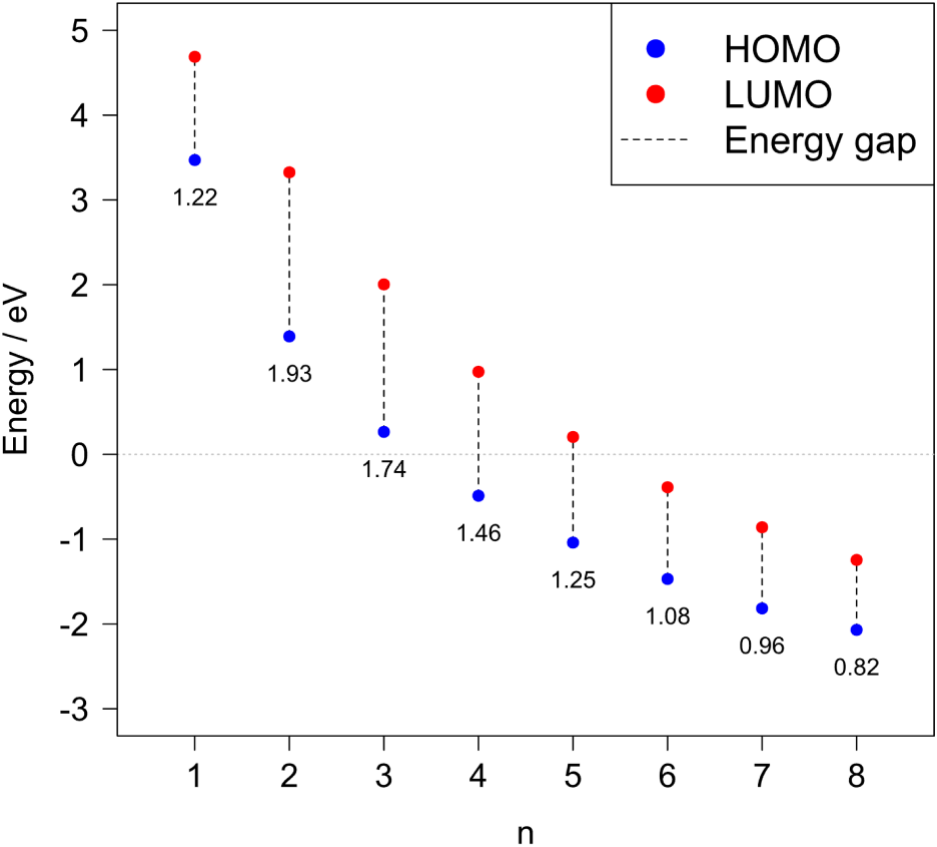
HOMO and LUMO energy levels for Chini clusters (*n* = 1–8). HOMO–LUMO energy gaps, indicated by the dashed lines, with specific annotated values, show a decreasing trend with an increasing cluster size, starting from the cluster with nuclearity equal to 2.

Next, the HOMO and LUMO energy levels for the Chini clusters (*n* = 1–8) were determined; see Figure 2 for plots of the HOMO and LUMO contours and Figure 3 for a schematic representation of the energy levels for Chini clusters (*n* = 1–8). For the clusters having *n* = 1–3, the HOMO energy level is above 0 eV, indicating that the electrons occupy the nonbonding orbitals. This may be reflected in their reactivity, making electron donation toward a reactant more likely. In contrast, as the cluster size increases, for *n* = 4–8, the HOMO energy levels drop below 0 eV.

The HOMO–LUMO energy gap decreases for *n* = 2–8 (see Figure 3), which is consistent with a redshift observed in the calculated UV–vis absorption spectra (see Section 3.3). The decreasing energy gap in larger clusters also points toward enhanced electronic delocalization, which could affect the optical and electronic properties of these materials. Furthermore, it is generally accepted that the systems having a smaller HOMO–LUMO gap are usually more reactive in redox processes.

**Figure 4:**
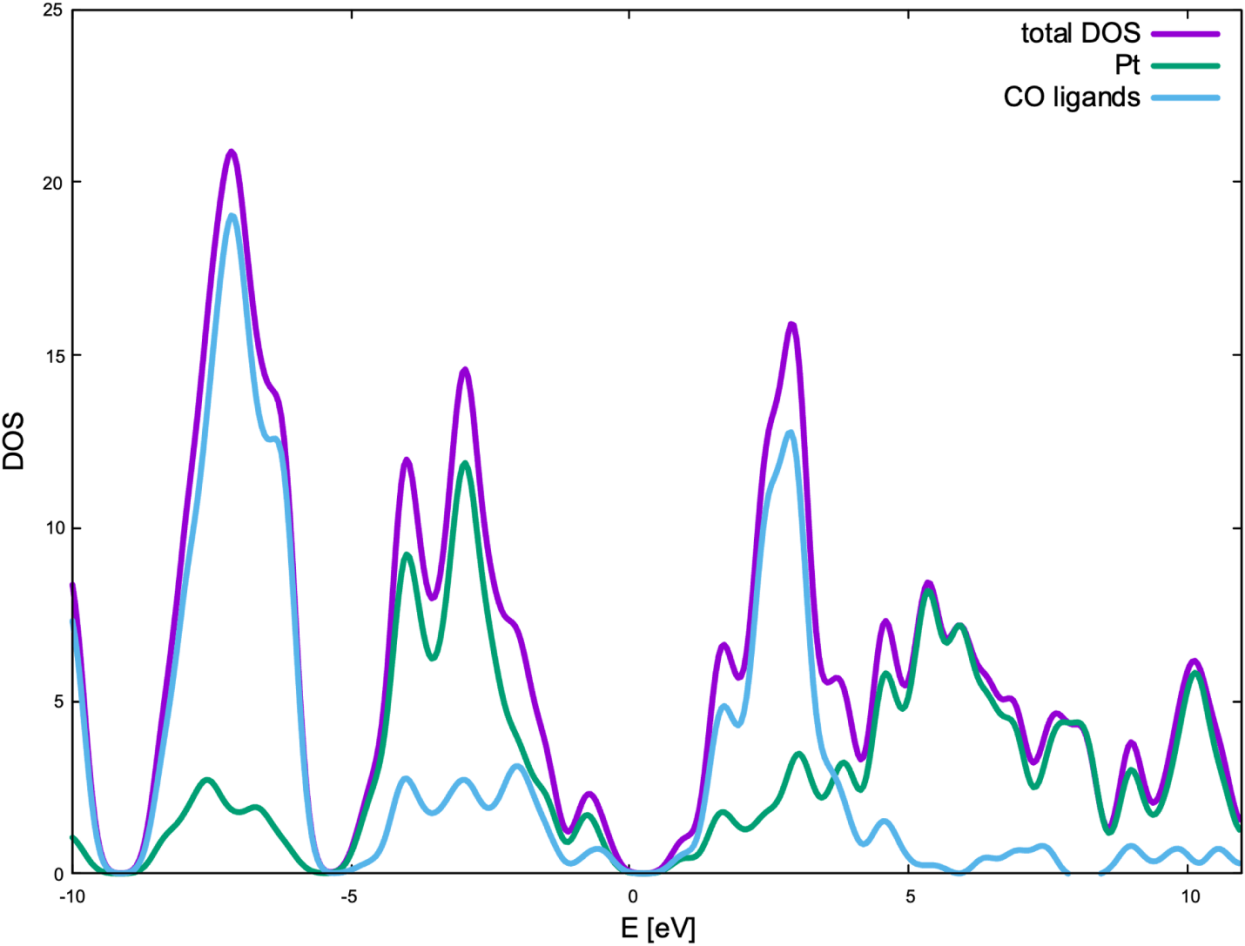
Plot of the density of states (DOS) for the Chini cluster with *n* = 4.

Furthermore, we have plotted the density of states (DOS) for the studied systems – see Figure S3. As we go from smaller clusters to the bigger ones, we clearly observe changes in the electronic structure of the systems. Initially, for systems with *n* = 1–2, the valence band of the cluster seems to be split into two regions – the broad peak spanning from ca. −6 eV to ca. −2 eV below the Fermi level, and the second, narrower band above ca. −1.5 eV. In the larger systems, these two bands become closer to one another. Figure 4 presents the partial DOS plot, where the contributions from the Pt atoms and the CO ligand are shown. The plot shows that the valence band is dominated by the orbitals of platinum atoms, while the conduction band is dominated by the orbitals originating from the CO ligands.

The observed trends in the HOMO and LUMO energy levels and energy gaps highlight that the electronic properties of the Chini clusters can be tuned by varying the cluster size. This feature is crucial for synthesizing clusters for specific applications, ranging from catalysis to electronic and optical devices.

### 3.3 Theoretical UV–vis Spectra

**Figure 5:**
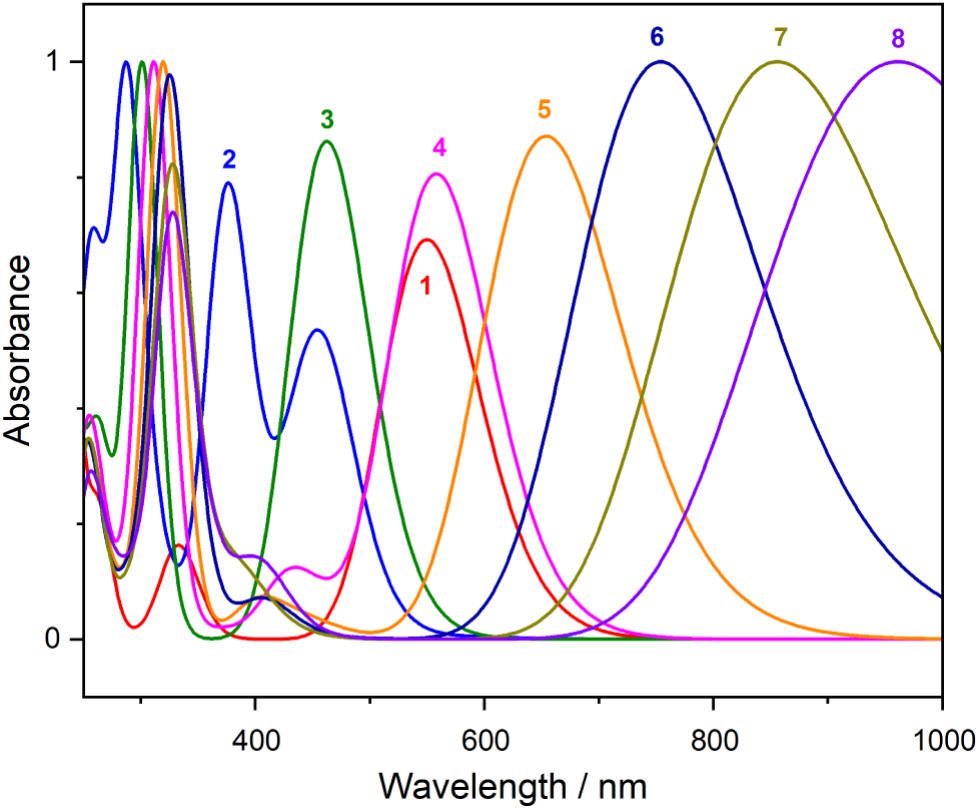
Simulated UV–vis absorption spectra of the Chini clusters for *n* = 1–8 calculated with the CAM-B3LYP hybrid functional in a vacuum. fwhm is set as 0.25 eV to imitate experimental broadness. Absorbance was normalized in a range from 0 to 1. Numbers above spectral lines indicate the nuclearity *n*.

The colors of the solutions containing Pt Chini clusters depend on their size (or nucleation number). The theoretical UV–vis absorption spectra for the Chini clusters (*n* = 1–8) were calculated—see Figure 5 for the simulated spectra.

Two distinct absorption peaks in the visible range of the spectrum are found. To assign the character of the electronic transitions responsible for such a spectrum, the character of the calculated transitions was analyzed for the Chini cluster with *n* = 3 – Figures S4 and S5. The broad, red-shifted band (lower-energy peak) corresponds to intertriangle transitions. These occur between different Pt_3_ units, involving interactions across the cluster. The narrow, higher-energy band is associated with intratriangle transitions, where electronic excitations involve CO orbitals within the same Pt_3_ unit. These transitions are more localized, higher-energy, and affect the transverse direction of the cluster.

Table 2 presents the main features of the broad red-shifted band of the Chini systems. Knowing the characteristics of the transitions and orbitals involved within, one can explain the significant redshift of the lower-energy absorption band (from 463 to 961 nm) observed for *n* = 3–8. The major contribution to these bands comes from the HOMO–LUMO excitation (see Table 2). As the size of Chini clusters increases, the excitation process primarily affects the electronic structure in the longitudinal (intertriangle) direction, while the transverse (intratriangle) one remains rather unaffected—the second absorption band shifts slightly from 350 to 370 nm. This anisotropic behavior in electronic excitation can be attributed to the spatial arrangement and interactions of Pt_3_ units within each cluster.

**Table 2:** Main Characteristics of the Lower-Energy Peak Corresponding to the Intertriangle Transitions

*n*	wavelength [nm]	oscillator strength	main contributions (in parentheses)
1	550	0.2352	HOMO–2 → LUMO (0.149)
			HOMO → LUMO+2 (0.671)
2	376	0.3880	HOMO → LUMO+1 (0.117)
			HOMO → LUMO+4 (0.626)
			HOMO → LUMO+8 (0.244)
3	463	0.5170	HOMO → LUMO (0.274)
			HOMO → LUMO+2 (0.186)
4	559	1.0414	HOMO → LUMO (0.474)
5	654	1.5080	HOMO → LUMO (0.476)
6	754	1.9648	HOMO–3 → LUMO+1 (0.011)
			HOMO → LUMO (0.476)
7	856	2.4369	HOMO–3 → LUMO+1 (0.014)
			HOMO → LUMO (0.474)
8	961	2.9117	HOMO–3 → LUMO+1 (0.016)
			HOMO → LUMO (0.471)

The observed redshift is also consistent with the decrease in the HOMO–LUMO energy gap as the cluster size increases (see Section 3.2), indicating that smaller energy is required for electronic transitions. Furthermore, the changes observed in the UV–vis spectra are consistent with the surface plasmon resonance (SPR) effect, which is characteristic of metal nanoparticles. The SPR effect results from the collective oscillation of electrons in response to light, which is stronger for larger clusters due to increased electron delocalization and interactions between Pt_3_ units. This effect contributes to the significant redshift and broadening of the absorption bands as the cluster size increases.

Our computational results are consistent with the experimental UV–vis spectra and other theoretical studies found in the literature,^3,40^ regarding the presence of the two distinct absorption bands and the size dependence of the energy of the low-energy transition.

### 3.4 Charge Transport Properties

**Figure 6:**
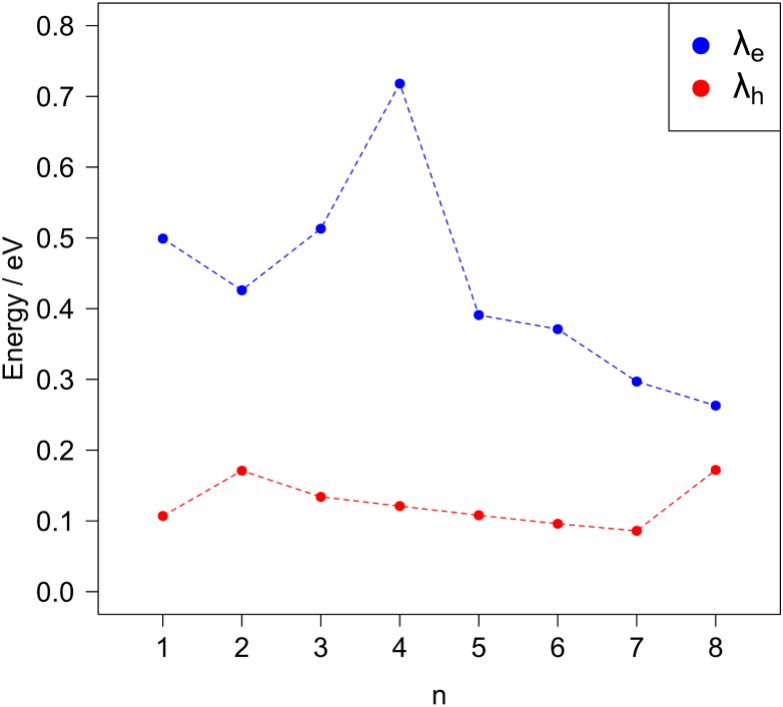
Calculated hole and electron reorganization energies for Chini clusters of different nuclearities. Dashed lines are employed for clarity and readability.

To describe the charge transport properties of the Pt Chini clusters, which are an important parameter of materials used in photocatalytic processes, hole and electron reorganization energies were determined using eqs 2 and 3 – see Figure 6. The reorganization energies are factors largely affecting the rate of the charge transfer. As per their definition (see eqs 2 and 3) they represent the changes of the geometry of the systems when an excess of positive or negative charge is introduced. Charge transfer reactions are associated with subtle or significant geometry modifications, such as changes in bond lengths and bond angles. Reorganization energies help in understanding these processes, as they represent energy losses associated with structural changes when an electron is donated or removed. They consist of internal reorganization energy, which involves changes within a molecule, and external reorganization energy, which involves changes in the surrounding solvent molecules. Here, the Chini clusters were modeled in a vacuum, so the external reorganization energies were neglected.

Higher reorganization energies typically result in slower charge transfer rates because more energy is required to overcome the energetic barriers associated with reorganizing the surrounding environment. Lower reorganization energies facilitate faster charge transfer processes. Based on our results, we can assume that the Chini clusters preferably act as hole transport materials (λ_h_ ≪ λ_e_). Notably, as the cluster size increases, electron transfer becomes more prevalent. Decreasing values of λ_e_ for larger clusters suggest that delocalization processes stabilize structures, leading to lower energy costs for electron transfer. For λ_h_, we observe fewer fluctuations in values compared to λ_e_ – all values are within the range of 0.1–0.2 eV. Increasing the size of clusters seems not to have a significant impact on the hole transfer. λ_h_ decreases for *n* = 2–7 but then increases for *n* = 8, showing irregular behavior.

The relatively small reorganization energy accompanying the changes in the number of electrons, especially for the structures with *n* > 4, also confirms that platinum Chini clusters can be employed as nanocapacitors or electron sinks in molecular and nanoelectronic devices.^5,41^

In the current paper, we decided not to evaluate the *V* parameter, which would characterize the orbital overlap between the neighboring platinum “triangles,” as the distance between them does not change significantly as we move from smaller to bigger clusters.

### 3.5 Theoretical Prediction of the Chini Clusters’ Activity in H_2_ Generation

The adsorption of the H_2_ molecule and H atom was investigated to predict the activity of the Chini clusters in H_2_ generation. The results are shown in Table 3 and Figure S6.

**Table 3:** Pt–H Bond Lengths in [Å] and Binding Energies (BE), Enthalpies (Δ*H*), and Gibbs Free Energies (Δ*G*) in [eV] for the H Atom and H_2_ Molecule Adsorption on the Chini Clusters with Varying Sizes (*n* = 1–8)^a^

	adsorption of H				adsorption of H_2_			
*n*	Pt–H [Å]	BE [eV]	Δ*H* [eV]	Δ*G* [eV]	Pt–H [Å]	BE [eV]	Δ*H* [eV]	Δ*G* [eV]
1	1.638	0.706	0.691	0.747	1.664–1.821	0.271	0.268	0.461
2	1.645	1.311	1.343	1.289	1.610–1.611	1.339	1.442	1.600
3	1.648	1.136	1.121	1.242	1.606–1.611	1.226	1.282	1.652
4	1.629	1.118	1.117	1.202	1.606–1.611	1.187	1.246	1.608
5	1.615	1.056	1.066	1.158	1.606–1.612	1.181	1.242	1.566
6	1.610	0.953	0.968	1.058	1.607–1.612	1.200	1.262	1.584
7	1.606	0.872	0.892	0.984	1.607–1.612	1.222	1.285	1.610
8	1.603	0.767	0.790	0.904	1.608–1.612	1.259	1.324	1.650

a Energies are calculated at PBE+D3/6-31G(d,p) for C, O, and H, and LANL2DZ for Pt.

The computed geometries of the adducts revealed specific sites on the clusters where hydrogen atoms prefer to bind. For the H atoms, we observe them bound to a single Pt center. The Pt–H distance ranges from 1.603 to 1.648 Å, consistent with the known values for Pt–H complexes.^42^ The H atom is positioned symmetrically, slightly moving toward the center of the triangle as the cluster increases, giving Pt–Pt–H angles that are similar in value, generally ca. 85–95°. An example of such a structure formed with [Pt_3_(CO)_6_]_4_^2–^ and the H atom is shown in Figure S7. Geometries of clusters bound with H atoms show slight distortions, particularly in smaller clusters. When the cluster size increases, the presence of more Pt_3_ units helps to stabilize the structures, minimizing the impact of H on their deformations.

Similarly, for the H_2_ molecules, both H atoms are coordinated to a single Pt center. Several attempts to obtain a cluster geometry with two H atoms on neighboring Pt centers were unsuccessful, except for the smallest cluster. Both Pt–H distances are similar in value, ranging from 1.607 to 1.821 Å, resulting in an H–H distance of 1.53 to 1.76 Å (the H–H distance in an isolated H_2_ molecule is 0.74 Å). The H_2_ ligand binding geometry indicates that its σ bonding orbital is involved in the coordination with the Pt center, in agreement with previous studies on H_2_ interaction with Pt surfaces.^43^ Again, an example of such an interaction is illustrated in Figure S7, taking [Pt_3_(CO)_6_–H_2_]_4_^2–^ as an example. Notably, for smaller clusters, significant structural deformations or even decomposition are observed during H_2_ bonding (the upper Pt_3_ unit moves farther apart from the rest of the cluster). The presence of more Pt_3_ units stabilizes the structures. However, such deformations could limit the effectiveness of these clusters and their stability as catalysts in hydrogen generation processes.

In addition, the energetic parameters accompanying hydrogen species binding (BE, Δ*H*, and Δ*G*) were analyzed. The observed trends indicate that the adsorption energies vary with the cluster size, with a maximum of all energetic parameters for clusters with *n* = 2–3 and showing a general decrease in all energetic parameters as the cluster size increases further. The positive values of Δ*G* indicate that the adsorption process is not thermodynamically favorable for the H_2_ molecules and the H atoms. The Chini clusters do not readily attract and retain these species on their surfaces. The desorption process, or the release of the H_2_ molecules from the Chini clusters, is thermodynamically favorable. This suggests that H_2_ is likely to desorb spontaneously when it forms on the surface of the cluster. That is the property that is desired from good candidates for H_2_ generation. We can also compare the energy parameters of hydrogen binding with those determined for different platinum surfaces. Depending on the Pt surface and the hydrogen coverage, the H binding energy lies in the range between −0.5 and +0.2 eV,^44,45^ showing almost energetically neutral adsorption. In the case of the studied clusters, the hydrogen binding is endothermic, indicating different behavior than pure metallic systems. This may lead to unique properties of the systems under study.

### 3.6 Experimental Verification

To verify theoretical predictions, the Chini clusters were synthesized under different pH conditions and deposited on TiO_2_, to check their activity in photocatalytic hydrogen generation. The nuclearity of the obtained systems was assigned using UV–vis spectroscopy: the samples synthesized at pH = 13 contain [Pt_3_(CO)_6_–H_2_]_4_^2–^, while those synthesized at pH = 7 contain [Pt_3_(CO)_6_–H_2_]_7_^2–^/[Pt_3_(CO)_6_–H_2_]_8_^2–^ – see Figure S8. It is worth mentioning that we did not obtain clusters smaller than *n* = 4.

**Figure 7:**
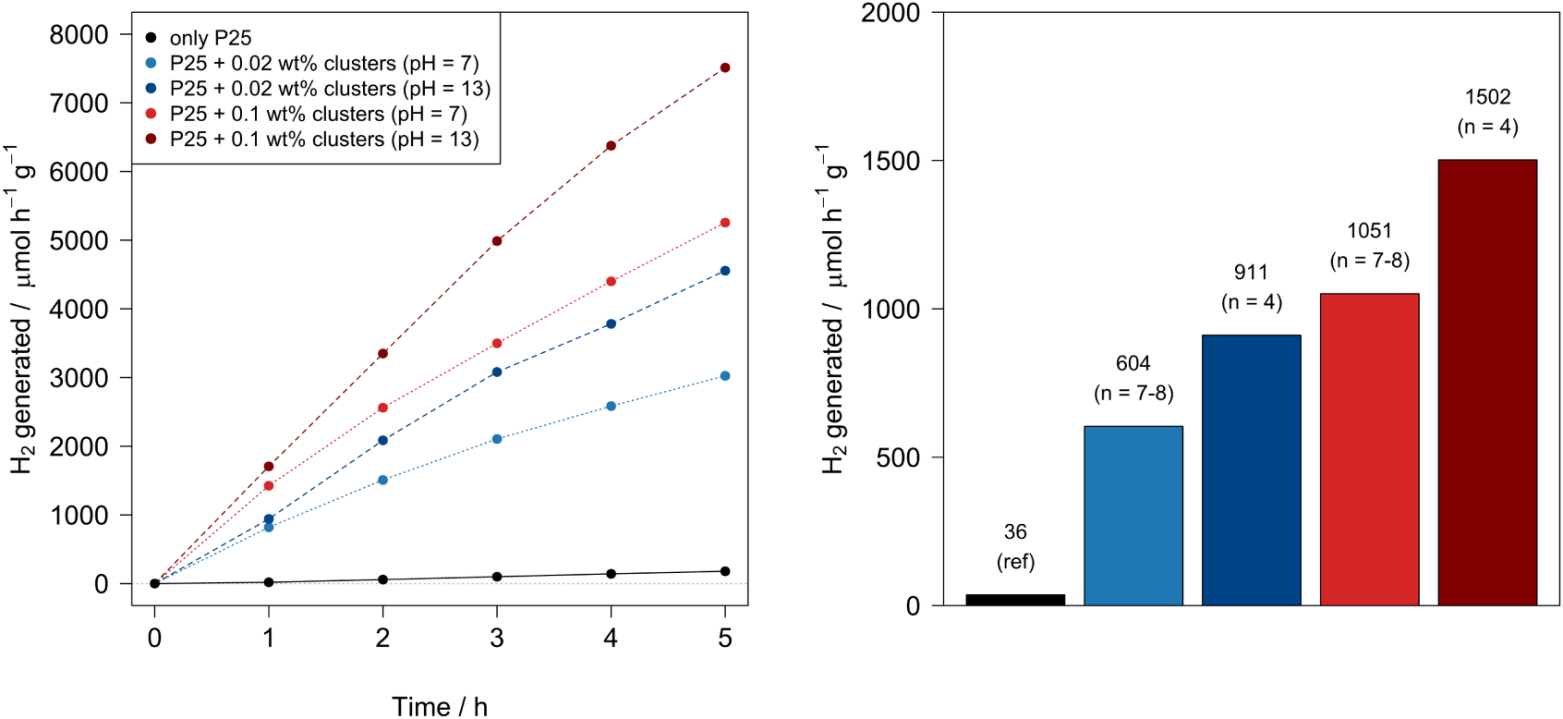
Photocatalytic H_2_ generation rates over time (left) and average H_2_ generation rates (right) for the Chini clusters impregnated on TiO_2_–P25. Red tones indicate samples with higher cluster content (0.1 wt % in platinum), while blue tones represent lower content (0.02 wt % in platinum). Darker tones and dashed lines correspond to higher pH (thus, lower nuclearity, assigned to *n* = 4), whereas lighter tones and dotted lines indicate lower pH, associated with larger clusters (*n* = 7–8).

Time-resolved microwave conductivity (TRMC) studies have shown that Pt Chini clusters very efficiently scavenge electrons from the CB of TiO_2_ decreasing the charge carriers’ recombination, which is beneficial for photocatalytic applications.^14^ The results of the photocatalytic experiments are shown in Figure 7. A substantial rise in the H_2_ generation activity is observed when the Chini–TiO_2_ composites are used instead of bare TiO_2_. This points out the activity of the Chini clusters in the H_2_ generation reaction. The analysis of the obtained data reveals two trends.

First, using photocatalysts with the Chini clusters obtained at higher pH conditions (pH = 13) leads to a higher rate of H_2_ generation. The Chini clusters synthesized under higher pH conditions (pH = 13) are smaller, with a size of *n* = 4, while those synthesized at neutral pH (pH = 7) are larger, with a size of *n* = 7–8. This confirms that smaller clusters formed at higher pH are more effective in the investigated catalytic processes.

Based on our calculations, we may hypothesize that the main geometric difference affecting the activity of the smaller clusters is the fact that they bind the hydrogen atom further apart from their surface. The Pt–H bond length is between 1.629 and 1.648 Å for the Chini clusters of *n* = 3–4, while this bond length is equal to 1.603–1.606 Å for clusters of *n* = 7–8. We also observe that the distance between the top Pt triangle (to which the H atom is attached) and the second Pt triangle is reduced as the size of the cluster is increased: it amounts to 3.133 Å in [H–Pt_3_(CO)_6_]_4_^2–^ and 2.983 Å in [H–Pt_3_(CO)_6_]_4_^2–^.

The positive values of Δ*H* and Δ*G* for adsorption of H_2_ suggest that the adsorption process is not thermodynamically favorable, implying that H_2_ is more likely to desorb spontaneously once formed on the surface of the cluster. Furthermore, higher values of Δ*H* and Δ*G* observed for smaller Chini clusters (*n* = 4) compared to the larger ones (*n* = 7–8) indicate that the desorption process is more likely to occur faster due to their higher reactivity. This characteristic is desirable for potential photocatalysts and may impact the H_2_ generation rates observed experimentally.

Similarly, differences between calculated electron and hole reorganization energies show that smaller Chini clusters can enhance charge separation more than larger ones. For the Chini clusters of *n* = 4, the difference is ca. 0.6 eV, while those of *n* = 8 show a lower difference (less than 0.1 eV). Charge separation is one of the critical factors impacting photocatalytic properties.

Second, a higher amount of Chini clusters leads to increased H_2_ generation. However, when the Pt amount in the samples is compared, the ones having lower Pt content show better performance. It is likely that the clusters are more effectively utilized, maximizing the available active sites on the TiO_2_ surface. Higher concentrations may lead to aggregation and other processes, reducing the overall catalytic efficiency per Pt atom.

Based on our data, including the DOS plots, as well as information from the literature, we think that the Chini clusters, when deposited on TiO_2_, modify its electronic state in a way similar to how metal nanoclusters do. Generally, photocatalytic H_2_ generation involves the following steps: photon absorption by the semiconductor, generation of the electron – hole pair, and their transfer toward the active sites at the surface, which are often present on the cocatalysts. There, the half-reactions leading to the production of O_2_ and H_2_ occur. Accordingly, the Chini clusters should be considered as cocatalysts at the surface of the titania, where protons acquire electrons and hydrogen is formed:
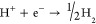


We also tried to model the reaction pathway on the basic [Pt_3_(CO)_6_]^2–^ unit. We considered binding of the second hydrogen on the adjacent Pt atoms and then the transfer of one of these toward the same Pt site where the first H sits. The process is accompanied by an energy barrier of 0.49 eV. Then, the H_2_ molecule can be formed, and its desorption is an exothermic process (0.271 eV). The scheme is depicted in Figure S9. For larger clusters we were unable to locate a structure with two H atoms on the adjacent Pt atoms.

The reported results are just proof of the DFT results presented above. The optimization of their performance, including the long-term stability of the Chini–TiO_2_ composites in the investigated process, requires further in-depth study.

## 4 Conclusions

The electronic structure analysis provides insights into the stability, reactivity, and potential applications of the Chini clusters, with size-dependent properties playing a leading role. Not only do the photochemical properties, such as the position of the electron absorption band, but also the electron and hole transport properties change in line with their nuclearity number *n*.

The reactivity of the Chini clusters toward the H and H_2_ species was also investigated. A direct indication of the origin of the higher activity of the smaller (*n* = 3–4) Chini clusters over the bigger ones (*n* = 7–8) is not straightforward. There can be many factors contributing to their superior performance, both geometric and electronic in nature. Our calculations point to the geometric differences in the Pt–H bond lengths and the intertriangle distances as the ones distinguishing the smaller Chini systems from the larger ones. It is, however, difficult to unanimously identify the main electronic factors determining the higher experimental activity of the smaller Chini clusters in H_2_ generation. Among the proposed ones, one can name the difference in the hole and electron reorganization energies, suggesting that the smaller Chini clusters can enhance charge separation more than the larger ones.

Our calculations indicate that the Chini systems may be considered components of the catalytic materials used for hydrogen generation. These assumptions were confirmed by testing the Chini–TiO_2_ composites in the photocatalytic H_2_ generation from a water–methanol mixture. The electronic, optical, and photocatalytic properties can be tuned by controlling the size of the metal cluster. Higher photocatalytic activity for H_2_ generation is obtained with the lowest nuclearity (*n* = 4 for experimental tests), in agreement with the DFT calculations.

## Supporting Information Available

Schematic representation of hole and electron reorganization energies for the Chini clusters (Figure S1); determined DFT geometries of the Chini clusters for *n* = 3–8 (Figure S2); DOS plots for the studied Chini clusters (Figure S3); the UV–vis absorption spectrum of [Pt_3_(CO)_6_]_3_^2–^ (Figure S4); visual representation of the orbitals among which the strongest transitions are observed for [Pt_3_(CO)_6_]_3_^2–^ in the UV–vis spectrum (Figure S5); calculated binding energies (BE), enthalpies (Δ*H*), and Gibbs free energies (Δ*G*) for the adsorption of hydrogen species on Chini clusters (Figure S6); the [Pt_3_(CO)_6_]_4_^2–^ cluster with the adsorbed H atom and the H_2_ molecule (Figure S7); experimental UV–visible spectra of Chini clusters synthesized in ethanol from 10^–3^ M Pt(acac)_2_ solution with different pH conditions (Figure S8); the proposed key step of H_2_ generation from two hydrogen atoms (Figure S9) (PDF)
