# Supplementary material for: Platinum Carbonyl Chini Clusters as Catalysts for Photocatalytic H2 Generation
Source: J Phys Chem C Nanomater Interfaces. 2025 Apr 23;129(17):8011–20. doi: 10.1021/acs.jpcc.5c00212 (PMC12051455; doi:10.1021/acs.jpcc.5c00212)
Supplement: Supplementary file 1 — jp5c00212_si_001.pdf [file jp5c00212_si_001.pdf]

Supplementary information file for:

Platinum Carbonyl Chini Clusters as Catalysts for Photocatalytic H_2 Generation

Aleksander Senderowski[1], Ana Andrea Méndez-Medrano[2], Isabelle Lampre[2], Hynd Remita*[2], Dorota Rutkowska-Zbik*[3]

[1]Interdisciplinary Centre for Mathematical and Computational Modelling, University of Warsaw, ul. Adolfa Pawińskiego 5A, 02-106 Warsaw, Poland
[2]Institut de Chimie Physique, UMR 8000 CNRS, Université Paris-Saclay, 91405 Orsay, France
[3]Jerzy Haber Institute of Catalysis and Surface Chemistry PAS, ul. Niezapominajek 8, 30-239 Kraków, Poland
e-mail: hynd.remita@cnrs.fr; ORCID: 0000-0003-3698-9327
e-mail: dorota.rutkowska-zbik@ikifp.edu.pl; ORCID: 0000-0001-9323-1710

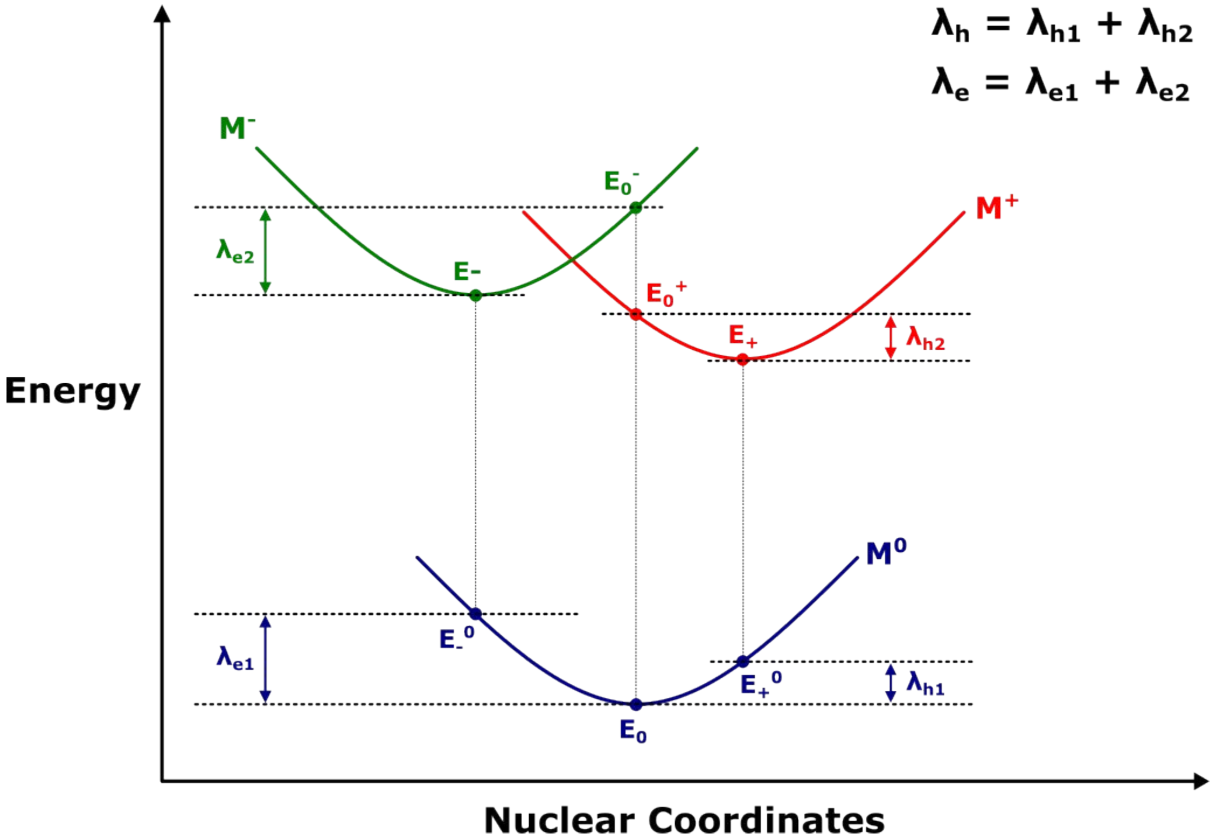

Figure S1. Schematic representation of hole and electron reorganization energies for the Chini clusters. The sequence of parabolas is based on energy levels calculated for a cluster with n = 4.

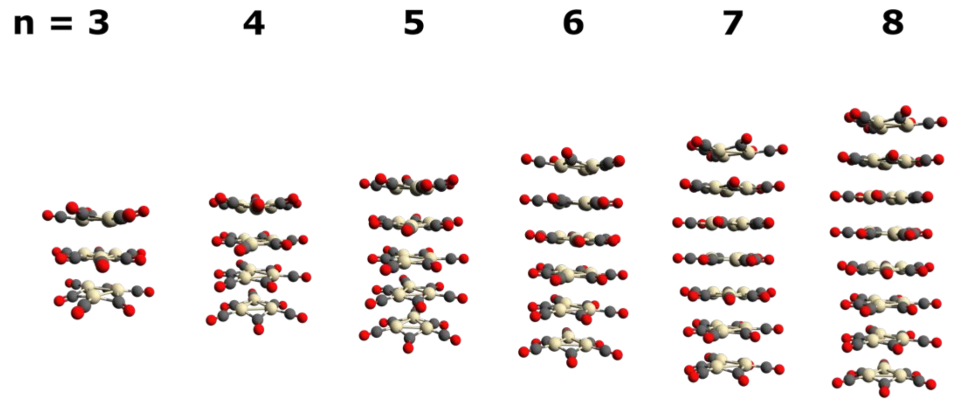

Figure S1. Determined DFT geometries of the Chini clusters for n=3-8.

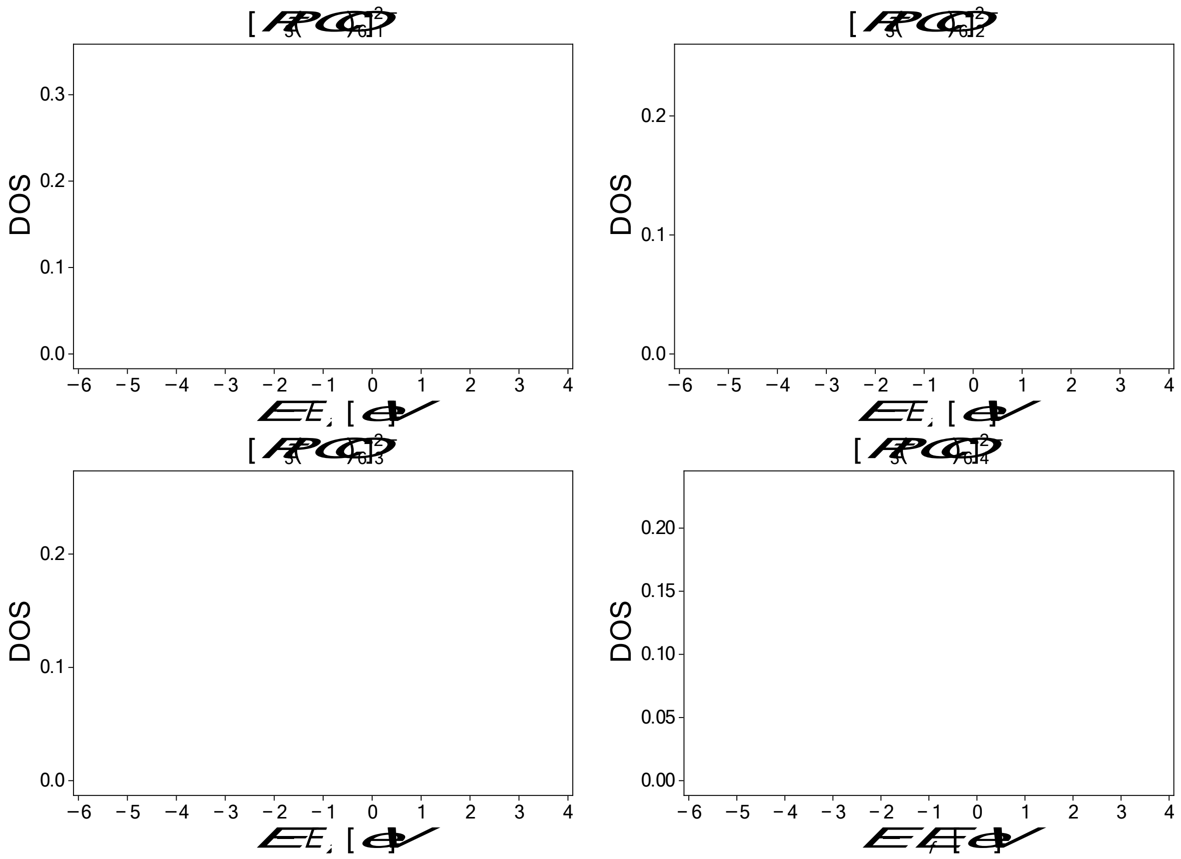

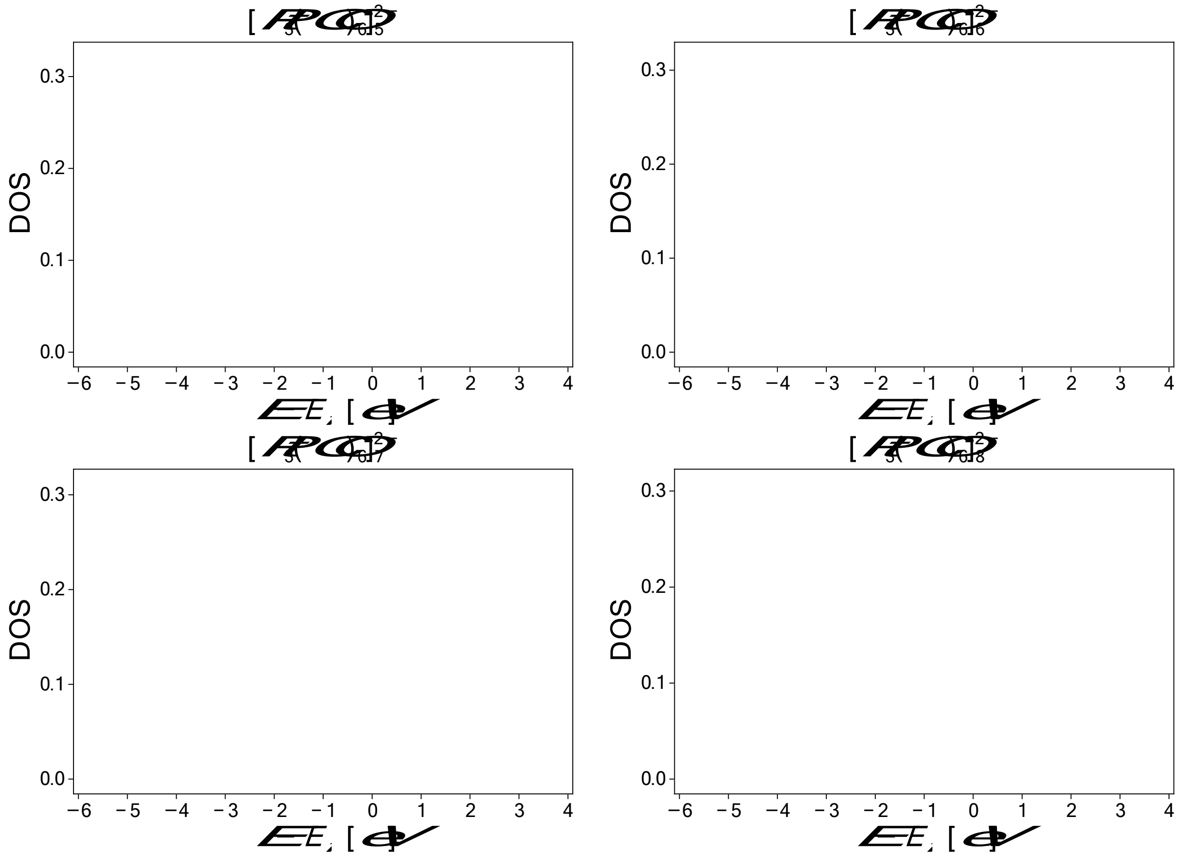

Figure S3. DOS plots for the studied Chini clusters.

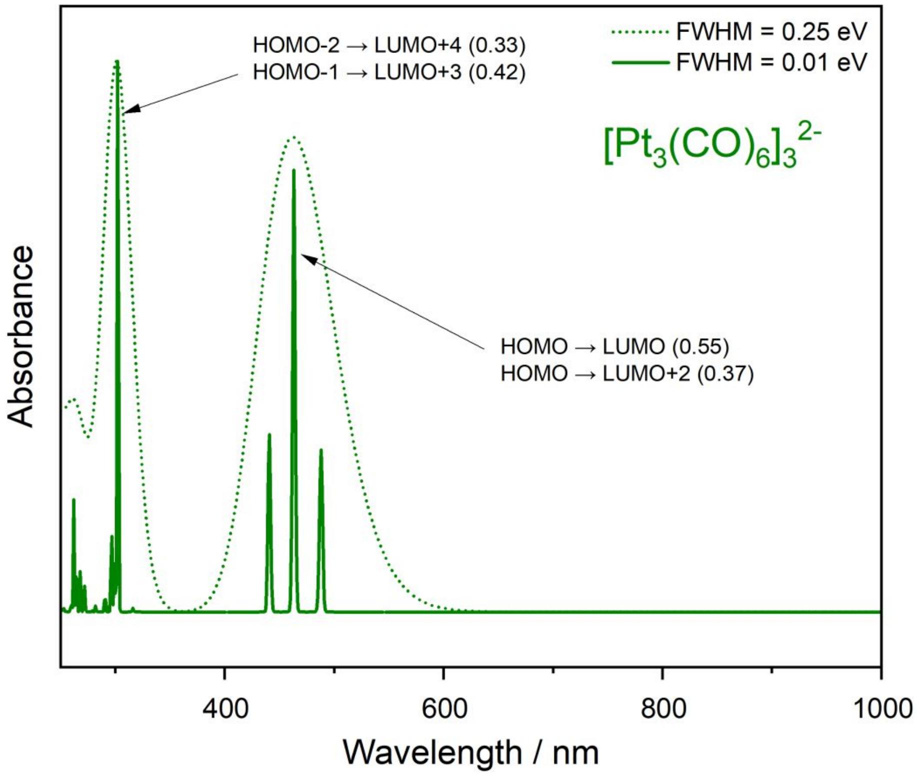

Figure S4. The UV-Vis absorption spectrum of $[Pt_3(CO)_6]_3^{2-}$. TD-DFT bands are presented as very narrow peaks (FWHM 0.01 eV, solid lines) to assign the occurring transitions. The broader spectrum (dotted lines) with FWHM set to 0.25 eV imitates experimental broadness. The specific transitions are labelled with the corresponding transitions and their contribution.

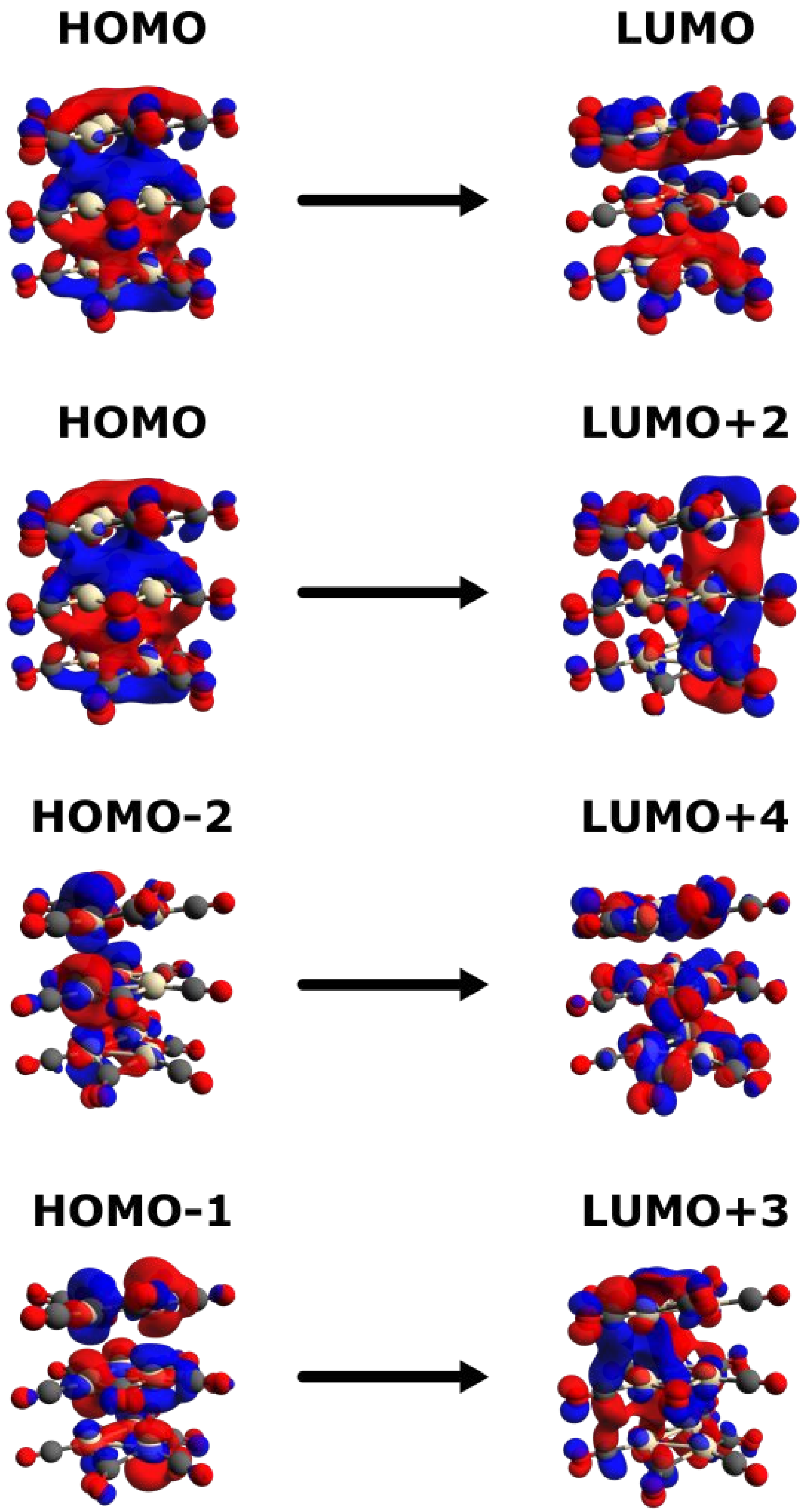

Figure S5. Visual representation of the orbitals among which the strongest transitions are observed for $[Pt_3(CO)_6]_3^{2-}$ in the UV-Vis spectrum.

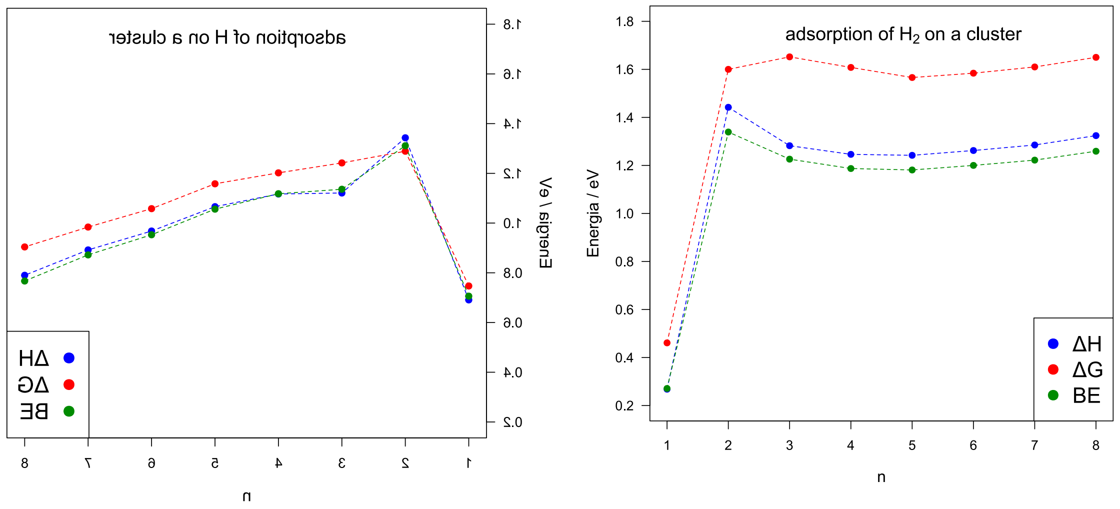

Figure S6. Calculated binding energies (BE), enthalpies (ΔH), and Gibbs free energies (ΔG) for the adsorption of hydrogen species on Chini clusters: the single H atom adsorption (left), the H_2 molecule adsorption (right).)

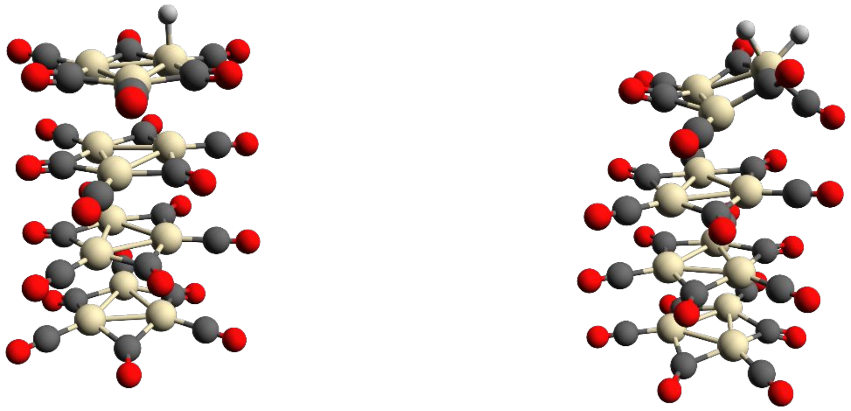

Figure S7. The $[Pt_3(CO)_6]_4^{2-}$ cluster with the adsorbed H atom (left) and the H_2 molecule (right). A significant distortion of the upper Pt_3 unit is observed in the geometry when H_2 is bound.

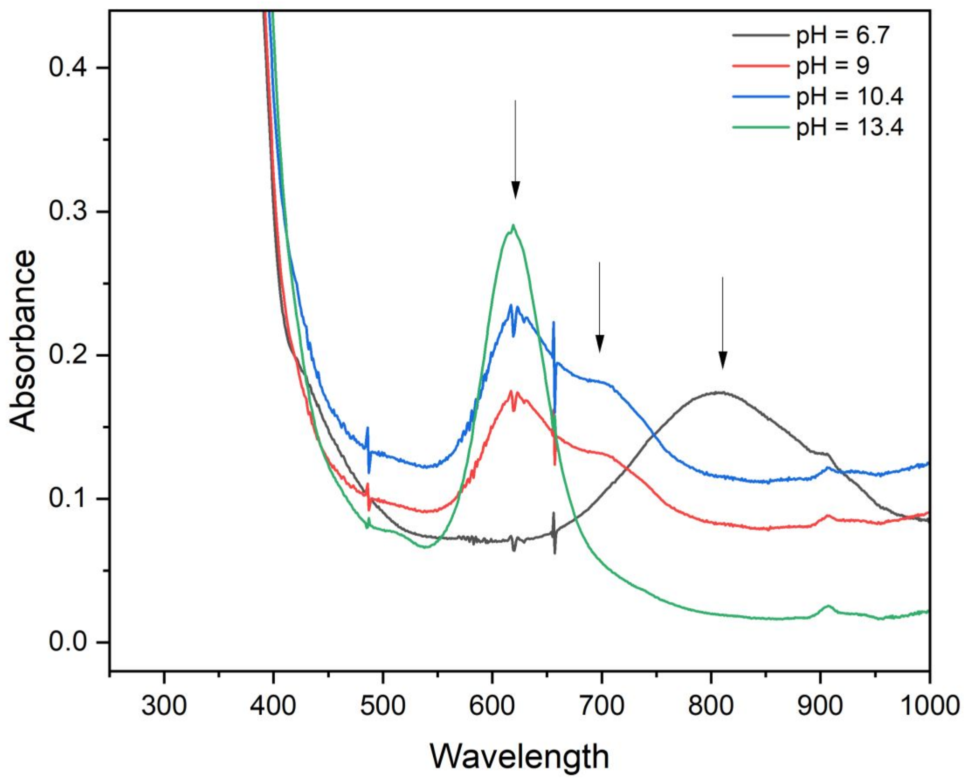

Figure S8. Experimental UV-Visible spectra of Chini clusters synthesised in ethanol from 10^{-3} M $Pt(acac)_2$ solution with different pH conditions (irradiation dose = 835(24) Gy; path length = 11cm).

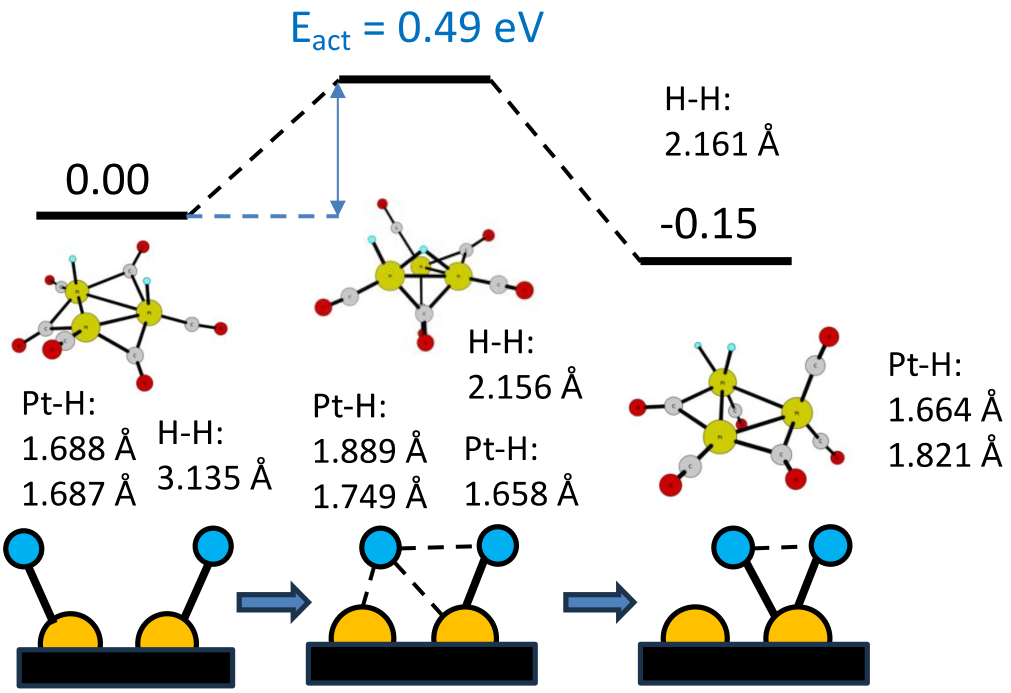

Figure S9. The proposed key step of the H_2 generation from two hydrogen atoms.
